# The tricky charm of the radial access

**DOI:** 10.1007/s12471-015-0748-8

**Published:** 2015-10-05

**Authors:** A. Azzano, P. Vermeersch

**Affiliations:** Cardiovascular Centre, Antwerp Hospital Network, Lindendreef 1, 2020 Antwerp, Belgium

Transradial access has now become a standard of care for percutaneous coronary angiography and interventions. We are currently dealing with a switch from transfemoral procedures towards transradial procedures because of several advantages related to the transradial catheterisation procedure, such as the lower number of access-related complications and early mobilisation. Recently, Valgimigli et al. suggested that radial access should become the default approach in patients at higher risk such as those with an acute coronary syndrome, with or without ST-segment elevation, undergoing invasive management. They observed that radial as compared with femoral access significantly reduces the rate of net adverse clinical events, defined as the composite of major adverse cardiovascular events or major bleeding. Moreover, the benefit of radial access is pronounced in highest-volume radial centres [1].

Radial artery occlusion (RAO) is considered the most common complication of the radial access, affecting 1.5–33 % of patients early after the procedure [2]. The true incidence could be underestimated because it is asymptomatic in the majority of cases. Nevertheless, a clinically palpable pulse does not confirm radial artery patency because it could be due to the collateral circulation. Moreover, non-occlusive radial artery injury as endothelial dysfunction, medial dissection and intimal hyperplasia may potentially also limit the use of the transradial route in future procedures. Therefore, for the transradial approach to remain a feasible access-site option in future percutaneous coronary interventions (PCI), the anatomical variation and mechanisms that underlie radial artery injury need to be better known and understood to minimise the risk of potential complications [2, 3]. Several procedural techniques, the refinement of material such as hydrophilic sheaths and smaller size equipment, and pharmacological treatments have led to a reduction in the rates of adverse events and have increased the therapeutic options, thus making the transradial percutaneous coronary procedure (TR-PCP) elegant, safe and feasible.

Zwaan, Koopman and colleagues provide a systematic review and meta-analysis about the current knowledge on the incidences of access-site complications and upper extremity dysfunction following TR-PCP. From a selection of 176 eligible papers, the authors reported a mean incidence of known complications of up to 9.6 %, which mainly consists of radial occlusion, radial spasm, swelling and haematoma. The incidence of upper extremity dysfunction after TR-PCP is up to 0.8 % even though it is rarely or not sufficiently investigated. Therefore, this reported low incidence could be underestimated due to the under-reported data in the literature. Moreover, the incidence of complications is often published by centres with more experience in using the transradial approach, where there are usually less access-site complications [4].

Additionally, the reported meta-regression analyses showed a significant positive correlation between transradial PCI and compartment syndrome (*p* = 0.014), probably related to major bleeding caused by the higher dosage of anticoagulants during PCI. Furthermore, the trend is not beneficial for early radial occlusion (4.9 %) and transradial coronary artery angiography (*p* = 0.060), as an effect of multiple catheter switches [4].

The authors emphasised that access-site complications might have a direct relationship with upper extremity dysfunction. Although few data have been reported about it, the authors have hypothesised that transitory ischaemia of the nerve due to radial spasm and irreversible nerve damage with a potentially debilitating outcome could occur. So, early detection and referral for any complications could be important to reduce their effect on upper limb function [4].

Despite the above-mentioned accurate analysis, it is interesting to consider what Van Leeuwen and colleagues recently reported. They investigated the effect of the transradial procedure on upper limb function demonstrating that there was no significant difference between radial and femoral access with respect to upper limb function, cold intolerance, or other procedure-related extremity complaints. It is an important finding, especially for those patients for whom optimal upper extremity function is essential [5].

Nevertheless, a limitation of the radial approach is still RAO, which may become symptomatic in some patients*.* Moreover, RAO may reduce the opportunities to use transradial access in further procedures, losing the advantage of the radial artery as a superficial access site for cardiac catheterisation [5].

The ability to recognise and treat complications of radial access is part of radial practice. It would be opportune that RAO is assessed at discharge and during follow-up, preferably by ultrasound. If unnoticed, complications could cause severe disability, with possible significant socioeconomic consequences.

Imaging data detected by optical coherence tomography and intravascular ultrasound as well as histological and functional studies have demonstrated significant structural and functional changes to the radial artery post transradial catheterisation, resulting in significant endothelial cell dysfunction. Minimising radial injury and occlusion should be an important component of transradial procedures and consists of endothelial protection, minimisation of endothelial wall trauma and minimisation of thrombus formation. Therefore, radial injury may be minimised through the use of smaller diameter guide catheters or through the use of sheathless guide catheters, reduction of catheter exchanges and minimisation of radial spasm periprocedurally, either through the use of spasmolytic drugs or through the use of coated sheaths/guide catheters. Thrombus formation within radial artery post transradial procedures may be reduced by routine anticoagulation with heparin periprocedurally and with non-occlusive radial artery compression postprocedurally [3].

The growing experience of transradial procedures and interventions is associated with a lower rate of access-site bleeding and vascular complications, improving clinical outcomes. Therefore the radial artery is an advantageous site of access and to use it in a safe way it is necessary to know its anatomy variability, the physiology and the eventual complications.

## Funding

None.

## Conflict of interests

None declared.
